# When one size does not fit all: aid and health system strengthening for Small Island Developing States

**DOI:** 10.1093/heapol/czad089

**Published:** 2024-01-23

**Authors:** Bruno Meessen, Anne Ancia, Danny Gill, Althea LaFoucade, Stanley Lalta, Guillermo Sandoval, Gade Waqa

**Affiliations:** Department of Health Financing and Economics, World Health Organization, 20 Avenue Appia, Geneva 1211, Switzerland; Mauritius Country Office, World Health Organization, Anglo Mauritius House Intendence Street, Port Louis, Mauritius; Ministry of Health and Wellness, Frank Walcott Building, Culloden Road, St. Michael BB11114, Barbados; Center for Health Economics & Department of Economics, University of the West Indies, 25A Warner Street, St Augustine 00000, Trinidad and Tobago; Centre for Health Economics, University of the West Indies, 25A Warner Street, St Augustine 00000, Trinidad and Tobago; Caribbean Subregional Program Coordination, Pan American Health Organization -World Health Organization, Dayrells and Navy Garden Roads, Bridgetown, Barbados; Pacific Research Centre for the Prevention of Obesity and Non-Communicable Diseases (C-POND), Fiji Institute of Pacific Health Research, Fiji National University, Tamavua Campus, Suva, Fiji

**Keywords:** Health systems, overseas development assistance, disaster

## Abstract

Health System strengthening is high on the agenda of the global health community. We review some of the specific challenges faced by Small Island Developing States in the development of their health systems. We propose a list of action points for aid actors willing to adapt their health programs and interventions.

Key messages

- There has been limited attention to the fact that because of fixed costs, the effectiveness of health system strengthening interventions may depend on the size of the health system to be strengthened. The burden on small countries is greater than the one on their bigger counterparts.
- Small Island Developing States (SIDS) face specific challenges in the development of their health systems, and this is true across the six building blocks of the health system.
- Development and aid actors, including multilateral agencies and developing banks, must pay more attention to the systemic constraints faced by SIDS and adapt their health programmes and interventions.

## Background

The World Health Organization (WHO) defines Health System Strengthening (HSS) as ‘any array of initiatives that improves one or more of the functions of the health systems and that leads to better health through improvements in access, coverage, quality or efficiency’ (WHO, 2019). HSS is now a concern across nearly all interventions, frameworks and policy guidance provided by WHO and other aid and development actors, including agencies with a more vertical mandate. The agenda also benefits from strong support at the level of ministries of health and academia.

Yet, as highlighted by Witter and colleagues (Witter *et al.*, 2019), knowledge gaps persist in the understanding and conceptualization of HSS. To our knowledge, there has been limited attention to the fact that the effectiveness and even the feasibility of HSS interventions may depend on the size of the health system to be strengthened. The delivery of some HSS interventions may entail substantial fixed costs or require costly complementary inputs such as a range of skills. This may impose greater burden on small countries than on their bigger counterparts and deprive the former of the returns to scale inherent to these interventions.

This commentary is a plea to the global health community to pay more attention to the adverse constraints encountered by small countries and more specifically Small Island Developing States (SIDS) in the development of their health systems. In this commentary, we review some of the specific challenges faced by SIDS regarding HSS and identify possible actions by the global health community.

## The reality of SIDS

Small Island countries face specific social, economic and environmental challenges. This reality was recognized as a special case at the 1992 United Nations (UN) Conference on Environment and Development in Rio de Janeiro. Accordingly, under the label of SIDS, the UN brought a distinct group of UN member states and non-UN members/associate members to benefit from a specific programme of action (Box 1).

Challenges faced by SIDS are structural and multi-faceted. Their remote geography and tropical climate have often influenced the settlement of human population, economic development and integration into the global economy. History left its marks, especially for islands integrated into the triangulated trade routes during colonial times. Today, location still entails irregular international traffic volumes and thus high import and export costs for goods. For many goods, SIDS rely on external markets, often with a limited number of importers. The reliance on imported processed food also has implications on nutrition and non-communicable diseases (NCD) (Hickey and Unwin, 2020; Guell *et al.*, 2022). The often very small population of SIDS does not allow them to seize economies of scale and be readily diversified. Many SIDS depend on a small pool of commodity exports as well as the service sector, particularly tourism, and are therefore highly vulnerable to economic shocks (UNEP, 2014). This was evidenced by how COVID-19 brought the tourism industry to a halt in many of them.

Box 1. The United Nations’ list of SIDS

The 39 States: Antigua and Barbuda, Bahamas, Barbados, Belize, Cabo Verde, Comoros, Cook Islands, Cuba, Dominica, Dominican Republic, Fiji, Grenada, Guinea-Bissau, Guyana, Haiti, Jamaica, Kiribati, Maldives, Marshall Islands, Federated States of Micronesia, Mauritius, Nauru, Niue, Palau, Papua New Guinea, Samoa, São Tomé and Príncipe, Singapore, St. Kitts and Nevis, St. Lucia, St. Vincent and the Grenadines, Seychelles, Solomon Islands, Suriname, Timor-Leste, Tonga, Trinidad and Tobago, Tuvalu, Vanuatu.

The 18 associate members of United Nations Regional Commissions: American Samoa, Anguilla, Aruba, Bermuda, British Virgin Islands, Curacao, Cayman Islands, Commonwealth of Northern Marianas, French Polynesia, Guadeloupe, Guam, Martinique, Montserrat, New Caledonia, Puerto Rico, Sint Maarten, Tusks and Caicos Islands, U.S. Virgin Islands.

Each SIDS is a particular case, depending on its size, geographical location, neighbours, etc. Some are also in the United Nations’ list of least developed countries; others are high-income countries. This paper focuses on those which receive international aid assistance for the strengthening of their health systems. However, some challenges, especially those stemming from the impossibility to benefit from economies of scale, may also apply to small Island territories (‘associate members’).

Climate change has a tangible impact on SIDS. Sea level rise and unpredictable climatic events such as heavy rainfalls and drought, which impact crops, prevalence of vector borne diseases and availability of clean water, pose an existential threat to some communities. Hurricanes, for example, destroy communication, energy and transport infrastructure, homes, schools and health facilities; as shown by some recent research, storms even impact the political system (Rahman *et al.*, 2022). The smallness of territory also implies that natural shocks are often systemic. An earthquake, a volcano eruption, a tsunami or a hurricane will disrupt every aspect of human activity limiting the country’s ability to buffer shocks at community and/or national level. Preparedness, response and adaptation will also be constrained by limited institutional capacity and scarce financial resources.

SIDS have of course their own strengths. As ‘paradisiac islands’, many have managed to become attractive touristic destinations. Holding vast territorial waters is another asset. Several of them, by their location, have geostrategic value which creates some negotiation power. Moreover, some countries are categorized as upper-middle or high-income. Although, with SIDS, many of our standard concepts, including the GDP/capita metric, deserves scrutiny: being ‘graduated’ by the World Bank or GAVI is not necessarily a blessing, given the structural vulnerability of SIDS economies to shocks, as shown again by the COVID-19 pandemic or the recent inflation crisis.

There is growing recognition of these specific vulnerabilities by the development community. For instance, with the support of its partners, the government of Barbados has introduced natural disaster clauses (Ho and Fontana, 2021) and more recently a pandemic clause, in its issuance of bonds. This recognition is also taking place among health aid actors. WHO regional offices have established specific subregional programmes, secretariats or divisions. A SIDS summit was held in WHO Geneva in June 2021 and another SIDS high-level meeting on NCDs and mental health was held in January 2023 followed by a SIDS Ministerial Conference in Barbados in June 2023.

## SIDS health systems through the lens of the six building blocks

WHO’s *‘Everybody’s business strengthening health systems to improve health outcomes*’ 2007 report has popularized the HSS building blocks framework (WHO, 2007). Although this framework has its limitations (Belghiti Alaoui *et al.*, 2020), the six building blocks are still comprehensive enough to guide a review of constraints faced by SIDS.

### Health system governance

Many governance functions—e.g. issuance of new legislation or setting priorities—entail some fixed costs: a minimal amount of resources, including time, is needed, whatever the size of the country. Analysing the competition on time of national cadres, Belghiti *et al.* have argued that HSS requires to manage simultaneously five dynamics: the services, the programmatic, the political, the reform and the capacity-building dynamics (Belghiti Alaoui *et al.*, 2020). In many SIDS, the Ministry of Health is a rather small team. Programmatic requests coming from global health actors may crowd out action on some of the other four dynamics of HSS. The limitation of the national knowledge ecosystem will also be constraining. In Sao Tome and Principe for instance, there is no faculty of medicine, research centre or consulting firm. This is a major constraint for building a learning health system—a requirement which has been increasingly identified as a key component of HSS (Sheikh and Abimbola, 2021). The Caribbean countries are in a better position, as they have managed to establish a regional knowledge ecosystem, with a central role played by The University of West Indies (with three of its research centres becoming WHO Collaborating Centers, in the areas of health economics and financing, nursing and midwifery development, and nursing policies and leadership).

### Health financing

The small size of the population and thus limited tertiary care facilities mean that highly technical medical care is limited in most SIDS; this entails a certain need for evacuations and health care abroad. Besides the overall burden on households (often through out-of-pocket payments), the economy and the trade balance, from a HSS perspective, this means that specific health financing agreements with some bigger countries must be established. There is no international guidance on how to develop such schemes; yet, for these countries, such agreements are an important contribution to universal health coverage.

### Human resources for health

Sustaining the supply, recruitment and retention of human resources for health is a problem faced by many SIDS. Problems already appear in the education stage: many SIDS rely on other countries for the education of several categories of their health personnel. The brain drain weighs on the performance of health facilities, the health system and the economy (De la Croix *et al.*, 2014; Rolle Sands *et al.*, 2020). The paucity of ‘systemic’ expertise (e.g. epidemiologists, health economists) in-country is also a constraint, given that HSS is largely about handling growing complexity.

### Health information system

Like elsewhere, paper-based information systems create an unnecessary burden on scarce qualified resources. But digitalization is slow in SIDS. The insufficiently diversified economy and maybe a lower perceived need for professional digital solutions (given the ease to sustain face-to-face interactions) deprive many SIDS from a vibrant digital ecosystem; yet, developers are key to tailoring and maintaining health information systems and digitalized patient files, even open-source solutions. This creates a dependence on foreign expertise. While equipping the few health facilities with computers and tablets will not be a major issue for donors providing support, this will usually not be enough. A related concern is the inadequacy of personnel to ‘research’ and ‘transform health data’ into usable evidence to assist in decision-making.

### Infrastructure, equipment and medicines

Isolation from trade routes, including limited airlift capabilities, small quantity purchased, and limited import competition together increase the costs of medicines, equipment and related products. Furthermore, most SIDS are too small to afford robust pharmaceutical control, regulatory systems and health technology assessments. The limited size of the national market also raises challenges for maintenance of equipment. When there are stock-outs or breakdown of equipment, these may impact the entire national health sector. Exposure to natural disasters can be a real issue for infrastructure. For example, in September 2017, hurricane Irma significantly damaged the only hospital on the Island of Barbuda, removing most of the roof, destroying the structure and significantly reducing the availability of medical services.

### Health service delivery

All the challenges listed with the above building blocks have some impact on the service delivery (Brizan-St. Martin *et al.*, 2023). In some SIDS, there is only one hospital. Every citizen understands its vital importance. Furthermore, the performance of the hospital has economic implications: by the security it brings, it may be a crucial requirement for the development of tourism (in SIDS, the case for a cross-sectoral approach to economic development is often stronger than in bigger countries; global health actors, because of their specialization, rarely grasp these issues). The foregoing exerts pressure upon politicians, which may lead them to meddle in hospital staff’s daily activities. The national focus on hospital services can affect both the supply, with insufficient public resources allocated to primary health care, and the demand, with households going straight to the nearby hospital. Still, because of the small size of the population, the hospital may struggle to perform some of its more specialized functions. Indeed, some advanced technologies, which may matter to retain some specialists, require high volume of use to be cost-effective. More worryingly, for many procedures, high volume is also a determinant of quality and outcomes of care (Sandoval *et al.*, 2018).

## Ways forward

HSS in SIDS is an agenda that deserves more attention from the global health community.

### Set up dedicated platforms at regional and global levels

Several UN agencies are already active on that front; a major initiative has been the establishment of specific subregional programmes, secretariats or divisions by WHO regional offices. But, given that many challenges are shared across regions, action at global level is also needed. A community of practice gathering HSS experts based in SIDS is one path that can be explored. Given the wide geographical distribution of SIDS across oceans, a lot of its work will have to be carried out through asynchronous digital means.

### Adapt rules and programmatic processes shaping health aid

It is up to the aid sector to adapt to SIDS reality, not the other way round. The GDP/capita metric is an unfair and inappropriate yardstick to decide aid conditions. First, if purchasing power parities are not applied, it disadvantages countries like SIDS which import basic commodities at a high price. Second, it is an absolute value ignorant of uncertainty; it denies that economies are not equal in terms of exposure to natural risks or opportunities to diversify their structure. A way forward would be to combine or replace it with a metric capturing risks—for instance, the multidimensional vulnerability index (Ram *et al.*, 2019). Similarly, multilateral and bilateral health agencies must collectively revise and simplify their programmatic processes, which too often overwhelm the few senior technicians of the Ministry of Health. How do we ensure that they really strengthen health systems (instead of weakening them by sustaining dependence or creating programme ‘silos’)? The need for cross-programmatic efficiency is not specific to SIDS (Sparkes *et al.*, 2017). One should therefore not exclude that alignments found among global health agencies supporting SIDS prove replicable in other countries as well. SIDS could actually be perfect settings to test, with limited budgetary implications, new tools, systems and practices inspired by a true logic of harmonization. An interim approach would be to set up a specific global initiative to support SIDS to meet procedural requirements put by aid actors.

### Mainstream SIDS reality into guidelines and practices

Authors of ‘universal’ policy guidelines or manuals could also be requested to include in their documents specific implications for SIDS. This tailoring could be developed in partnership with WHO collaborating centres based in SIDS. Aid and development actors should indeed aim at strengthening SIDS knowledge ecosystem by commissioning studies, training and think-tanks functions to local academic institutions, with specific attention to linguistic constraints. We invite the global health community to reflect on how its ways of doing things (conferences, languages, formats) set barriers to some. There are new paths to explore; for instance, countries like Cape Verde, Guinea-Bissau or Sao Tome and Principe, which suffer from the limited availability of global guidelines and expertise in Portuguese, could benefit from South–South cooperation in the form of technical assistance from Brazil.

### Implement SIDS-specific HSS interventions

In close coordination with SIDS, aid agencies should also identify HSS interventions of greater relevance in SIDS. Some will flow directly from the specific pattern of risks faced by many SIDS. Investment in disaster preparedness belongs to this category. Others will stem from the small size of SIDS. To specify the content of these interventions, one approach would be: (1) to review health system functions and sub-functions (Papanicolas *et al.*, 2022) and identify those for which return to scale are substantial; (2) among these, to identify those for which outsourcing to a bigger country or pooling among small countries are feasible options and (3) to specify how aid agencies can support these outsourcing or pooling—oftentimes, it will be about facilitating partnerships. For functions and sub-functions without much return to scale (e.g. primary care) or which cannot be outsourced or pooled (e.g. decision by health authorities), the HSS interventions may be the ‘classical’ ones.

The building of capacity for telemedicine for specialized care only available abroad (or on the main island) is an example of a possible HSS ‘outsourcing’ intervention; it looks a low-hanging fruit, which could be supported, for instance, by bilateral aid agencies. An example of support to pooling is the current assistance given by the WHO Regional Office for Africa to African SIDS for a pooled procurement of some of their medicines. An even bolder idea would be to assist SIDS to pool revenues for them to resemble large-country health financing systems—there is an initiative in the Caribbean that is following this approach with a focus on some particular conditions and services.

### Value knowledge content from and to SIDS

‘Classical’ HSS interventions can also be tailored to SIDS as for their content. For instance, global health agencies could support research and learning agenda on HSS across SIDS. As an illustration, the regulated public–private partnerships developed by Barbados and a few other Caribbean countries for the procurement and delivery of medicines for chronic disease patients deserves wider consideration, i.e. how they have enrolled private retail pharmacies to secure financial access for all to essential medicines. A comparative review of referral systems and health financing mechanisms for medical care abroad and their implication on national health expenditure is a good example of a question with high relevance for SIDS (Suzana *et al.*, 2018). Economic growth often comes with a need for more complex institutional arrangements; how should SIDS position themselves in terms of health sector reform questions? For instance, should SIDS try, as other countries, to set up new health agencies independent from the Ministry of Health… or should they favour simple models?

### Value the global relevance of SIDS

Developing effective HSS interventions for SIDS will directly benefit their population. The climate justice agenda puts it as a requirement stronger than ever. But SIDS also have much to offer to the global health community. For instance, the NCD wave will soon hit low-income countries; the implications of this for HSS are still to be fully understood. Several SIDS have successfully handled mother and child health and communicable diseases. Their operational desk is ‘cleaned’; this is fortunate, given that they are confronted with some of the highest burden of NCDs in the world. Developing health-in-all policies, action against commercial determinants and innovative health service delivery models for the management of chronic illnesses are top priorities for them (CARICOM, 2007; WHO, 2023). Committing attention and resources to their plans, actions and battle (Boseley, 2023) would generate huge learning benefits for the global health community at a low cost. Climate change reminds us that we are one world. Let us leave no one behind, but also value what smaller countries offer to us.

## Reflexivity statement

This paper is about the challenges faced by SIDS in building their health systems. Contributors 3, 4, 5 and 7 are SIDS nationals and have been active in health system strengthening in their region. Contributors 1, 2 and 6 are not SIDS nationals, but have been working in SIDS over the last years (long-term assignment for 2 and 6; short-term assignments for 1). All together as a group of authors, we have had exposure to reality of the Caribbean islands, Gulf of Guinea islands, Indian Ocean islands and Pacific islands. Our authorship also involves different knowledge holders: academia (4, 5, 7), Ministry of Health (3) and international agencies (the three levels of WHO: HQ, Regional Office and Country Office). Three authors are female and four are male.

## References

[R1] Belghiti Alaoui A, De Brouwere V, Meessen B, Bigdeli M. 2020. Decision-Making and health system strengthening: bringing time frames into perspective. *Health Policy and Planning* 35: 1254–61. 33450766 10.1093/heapol/czaa086 PMC7810387

[R2] Boseley S. Barbados fights Big Sugar for the survival of its people. The Guardian, https://www.theguardian.com/global-development/2023/jun/08/barbados-fights-big-sugar-for-survival-of-its-people-diabetes, accessed 12 August 2023.

[R3] Brizan-St. Martin R, St. Martin C, La Foucade A, Mori Sarti F, McLean R. 2023. An empirically validated framework for measuring patient’s acceptability of health care in Multi-Island Micro States. *Health Policy and Planning* 38: 464–73. 36760180 10.1093/heapol/czad012

[R4] CARICOM . 2007. Declaration of Port-of-Spain: uniting to stop the epidemic of chronic NCDs. Sept 16, 2007. https://caricom.org/declaration-of-port-of-spain-uniting-to-stop-the-epidemic-of-chronic-ncds/, accessed 12 February 2023.

[R5] De la Croix D Docquier F Schiff M . 2014. Brain drain and economic performance in small island developing states. In: Artal-Tur A, Peri G and Requena-Silvente F (eds). *The Socio-Economic Impact of Migraltion Flows*. Cham, Switzerland: Springer International Publishing, 123–44.

[R6] Guell C, Brown CR, Navunicagi OW et al. 2022. Perspectives on strengthening local food systems in Small Island Developing States. *Food Security* 14: 1227–40. 35528949 10.1007/s12571-022-01281-0 PMC9067893

[R7] Hickey G, Unwin N. 2020. Addressing the triple burden of malnutrition in the time of COVID-19 and climate change in Small Island Developing States: what role for improved local food production? *Food Security* 12: 831–5. 32837642 10.1007/s12571-020-01066-3 PMC7343409

[R8] Ho SJ, Fontana S. 2021. *Sovereign Debt Evolution: The Natural Disaster Clause*. 11, Clearly Gottlieb.

[R9] Papanicolas I, Rajan D, Karanikolos M, Soucat A, Figueras J (editors). 2022. *Health System Performance Assessment: A Framework for Policy Analysis*. Geneva: World Health Organization. Health Policy Series, No. 57. 37023239

[R10] Rahman MH, Anbarci N, Ulubaşoğlu MA. 2022. “Storm autocracies”: islands as natural experiments. *Journal of Development Economics* 159: 102982.

[R11] Ram J, Cotton J, Frederick R, Elliott W. 2019. Measuring vulnerability: a multidimensional vulnerability index for the Caribbean (No. 2019/01). CDB Working Paper.

[R12] Rolle Sands S, Ingraham K, Salami BO. 2020. Caribbean nurse migration—a scoping review. *Human Resources for Health* 18: 1–10. 32178696 10.1186/s12960-020-00466-y PMC7077100

[R13] Sandoval GA, Brown AD, Wodchis WP, Anderson GM. 2018. Adoption of high technology medical imaging and hospital quality and efficiency: towards a conceptual framework. *International Journal of Health Planning and Management* 33: e843–60. 10.1002/hpm.2547 29770971

[R14] Sheikh K, Abimbola S and World Health Organization. 2021. Learning health systems: pathways to progress: flagship report of the alliance for health policy and systems research.

[R15] Sparkes S, Durán A, Kutzin J. 2017. *A System-wide Approach to Analysing Efficiency across Health Programmes*. Geneva: World Health Organization.

[R16] Suzana M, Walls H, Smith R, Hanefeld J. 2018. Achieving universal health coverage in small island states: could importing health services provide a solution?. *BMJ Global Health* 3: e000612. 10.1136/bmjgh-2017-000612 PMC5841501 29527349

[R17] UNEP . 2014. *Emerging Issues for Small Island Developing States. Results of the UNEP/UN DESA Foresight Process*. Nairobi, Kenya: United Nations Environment PRogram (UNEP).

[R18] WHO . 2007. *Everybody’s Business—Strengthening Health Systems to Improve Health Outcomes: WHO’s Framework for Action*. Geneva, Switzerland: World Health Organization.

[R19] WHO . 2019. Health system glossary. https://www.who.int/docs/default-source/documents/health-systems-strengthening-glossary.pdf, accessed 7 November 2023.

[R20] Witter S, Palmer N, Balabanova D et al. 2019. Health system strengthening—reflections on its meaning, assessment, and our state of knowledge. *The International Journal of Health Planning and Management* 34: e1980–9. 31386232 10.1002/hpm.2882

[R21] WHO . 2023. 2023 Bridgetown Declaration on NCDs and mental health. https://cdn.who.int/media/docs/default-source/ncds/sids-event/2023-bridgetown-declaration-on-ncds-and-mental-health.pdf?sfvrsn=5feda33f_11, accessed 30 June 2023.

